# RT-4M: Real-Time Mosaicing Manager for Manual Microscopy System

**DOI:** 10.3390/s25102968

**Published:** 2025-05-08

**Authors:** Nobuhito Mori, Yoshihiro Miyazaki, Tatsuya Oda, Yasuyuki S. Kida

**Affiliations:** 1 Cellular and Molecular Biotechnology Research Institute, National Institute of Advanced Industrial Science and Technology (AIST), Tsukuba 305-8565, Ibaraki, Japan; 2 Department of Gastrointestinal and Hepato-Biliary-Pancreatic Surgery, Institute of Medicine, University of Tsukuba, Tsukuba 305-8575, Ibaraki, Japan; 3 School of Integrative and Global Majors, University of Tsukuba, Tsukuba 305-8575, Ibaraki, Japan

**Keywords:** whole slide imaging, virtual slide, stitching, digital pathology

## Abstract

The creation of virtual slides, i.e., high-resolution digital images of biological samples, is expensive, and existing manual methods often suffer from stitching errors and additional reimaging costs. To address these issues, we propose a real-time mosaicing manager for manual microscopy (RT-4M) that performs real-time stitching and allows users to preview slides during imaging using existing manual microscopy systems, thereby reducing the need for reimaging. We install it on two different microscopy systems, successfully creating virtual slides of hematoxylin and eosin- and fluorescent-stained tissues obtained from humans and mice. The fluorescent-stained tissues consist of two colors, requiring the manual switching of the filter and an exposure time of 1.6 s per color. Even in the case of the largest dataset in this study (over 900 images), the entire sample is captured without any omissions. Moreover, RT-4M exhibits a processing time of less than one second per registration, indicating that it does not hinder the user’s imaging workflow. Additionally, the composition process reduces the misalignment rate by a factor of 20 compared to existing software. We believe that the proposed software will prove useful in the fields of pathology and bio-research, particularly for facilities with relatively limited budgets.

## 1. Introduction

Virtual slides, also known as whole slide images, are high-resolution, wide-range digital images of biological samples, such as pathological specimens and cultured cells, that can be observed on a computer using a microscope as if they were real slides [1]. As virtual slides contain digitalized data, they are easier to store than the actual specimens themselves and less susceptible to degradation and damage. They are also useful in various applications, such as remote pathological diagnosis, artificial intelligence-assisted diagnosis, and the education of pathologists. Commonly used tools for creating virtual slides include slide scanners and automated microscopes, and the grid images obtained using automated microscopes can be stitched together using specialized software to create them as well [2,3,4,5]. However, the aforementioned precision instruments are expensive and unsuitable for hospitals or laboratories with limited budgets. A relatively inexpensive alternative involves capturing multiple images using a manual microscope while changing the field-of-view (FOV) accordingly and stitching them together subsequently using batch processing, which is referred to as the offline method in this study. In this method, different images are aligned based on overlaps between them using algorithms such as feature point extraction and phase correlation [6,7]. These techniques have continued to evolve in recent years, with one notable improvement being the use of globally consistent alignments selected from multiple registration candidates to address failure cases in repetitive or featureless regions [8]. Concurrently, comparative studies have also been conducted on various stitching techniques, including traditional feature-based and deep learning-based methods, highlighting their robustness and efficiency across different microscopy modalities [9]. Both free and proprietary software options are available for the offline method [10,11]. The use of proprietary software, originally designed for panoramic photo stitching rather than for microscopy, has also been explored for creating virtual slides [12,13]. The offline method software enables users to utilize their existing microscopy systems, facilitating the inexpensive integration of virtual slides into established workflows. However, as users cannot preview the stitched images during the sample imaging process, stitching errors or partial image loss may occur during stitching. In such cases, the sample must be reinstalled and realigned on the microscope, and the imaging conditions must be reset, or reimaging must be performed, which becomes significantly time- and effort-intensive. Alternatively, images may be dynamically read from the camera while moving the microscope manually for stitching—this is referred to as the on-memory real-time method in this study [14]. This method allows users to preview the virtual slide created during imaging, thereby reducing the need for reimaging. However, it requires dedicated software and cameras, hindering the use of existing image acquisition software and cameras. In addition, the mechanism for dynamic image reading from a camera is unsuitable for imaging fluorescent samples that require long exposure times or filter switching.

**Figure 1:**
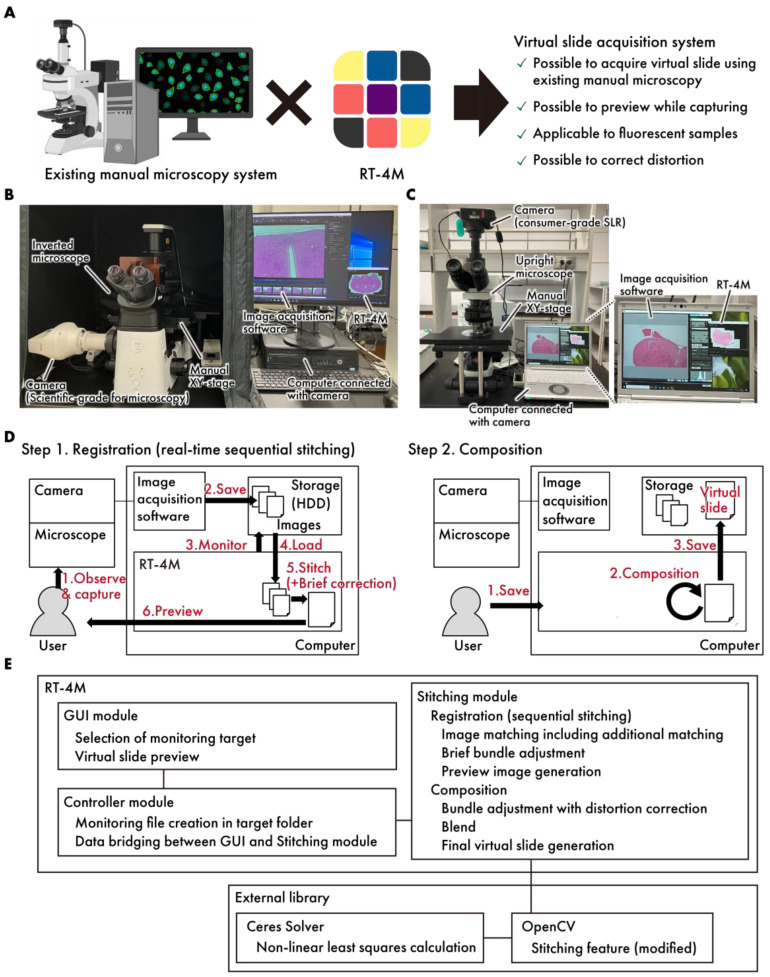
A concept diagram of RT-4M. (**A**) By installing RT-4M on an existing manual microscopy system, an environment for creating virtual slides can be established. (**B**,**C**) The actual system setup. The system allows for flexible configurations, such as (**B**), a setup where a microscope-dedicated camera is connected to a manual inverted microscope, and RT-4M is added to a computer equipped with image acquisition software for the camera, or (**C**), a setup where a consumer-grade SLR camera is connected to a manual upright microscope, and RT-4M is added to a computer with the utility software for the camera. (**D**) RT-4M comprises registration and composition stages. During the registration stage, it synchronizes with the user’s microscopy imaging, performing real-time image stitching and preview display. The composition stage enhances image quality and saves virtual slides. (**E**) The software architecture of RT-4M. RT-4M adopts a conventional Model–View–Controller (MVC) architecture, comprising a GUI module (view), a controller module (controller), and a stitching module (model). The major external libraries used in the system include OpenCV and Ceres Solver.

In this study, we propose a real-time mosaicing manager for manual microscopy (RT-4M), which performs the sequential stitching of images acquired via manual microscopy in real time and creates virtual slides on this basis (Figure 1A). This software is loosely coupled with an image acquisition software and a camera. In particular, it detects the writing of image files to storage, e.g., hard disk and solid-state drives, by the image acquisition software and creates virtual slides. Therefore, RT-4M can be implemented simply by adding it to a computer with existing image acquisition software—it does not require dedicated cameras, making its implementation inexpensive and enabling users to continue using familiar systems. By creating virtual slides in real time, users can preview the stitching status while capturing images, thereby avoiding the need for reimaging owing to missed shots. Additionally, RT-4M can be used to create virtual slides from fluorescent samples that require manual filter switching and long exposure times because image stitching is triggered by the file writing action of the image acquisition software. It also corrects image distortion induced by the optical system automatically, enabling the creation of high-resolution virtual slides required for digital pathology. Thus, RT-4M is useful for creating virtual slides in hospitals and laboratories with limited budgets. Furthermore, it could potentially support downstream intelligent diagnostic and analytical tasks, which are becoming increasingly important in the context of computational pathology [15]. Integrating high-quality virtual slide data with machine learning frameworks enables advanced applications such as disease classification and grading [16,17], making systems like RT-4M a valuable foundation for future intelligent diagnostic pipelines.

## 2. Materials and Methods

### 2.1. System Architecture

The system architecture of RT-4M is illustrated in Figure 1B–E. The system consists of a microscope equipped with a camera and a computer. Image acquisition software and RT-4M were preinstalled on the computer. RT-4M operates in two stages—registration and composition. During the registration stage, the user performs imaging, and real-time virtual slide creation (sequential stitching) is executed accordingly. This process is designed to balance processing speed, which influences the user’s imaging operation, and image quality, which facilitates virtual slide creation. In particular, the user observes the sample under the microscope and captures images using the image acquisition software. When the image acquisition software saves image files to storage, RT-4M detects the file creation action, reads the images automatically, performs stitching with the previously created virtual slide using brief corrections, and displays a preview to the user. The user confirms whether the virtual slide is being created as intended based on the preview and adjusts the microscope’s FOV to overlap with the previously captured FOV before capturing the next image. By repeating this process, the desired range of the sample can be covered to create a virtual slide. During the composition stage, RT-4M enhances the quality of the virtual slide created during the registration stage. This involves minimizing the errors between images (bundle adjustment), correcting distortions, calculating seams, blending, and saving the virtual slide, all of which are computationally intensive tasks. In particular, when the user instructs RT-4M to save the virtual slide, requisite processing is performed to enhance image quality, and the virtual slide is saved to storage. By dividing the process into the registration and composition stages, RT-4M achieves both the real-time creation of virtual slides during the user’s imaging action and the high quality of the final output. Additionally, RT-4M is equipped with a loosely coupled architecture, performing one-way data communication via storage with the camera and image acquisition software, making it compatible with various types of cameras and image acquisition software. RT-4M (2.1.4) adopts a conventional Model–View–Controller (MVC) architecture, comprising a GUI module (view), a controller module (controller), and a stitching module (model). The GUI module provides the user with functionalities such as selecting the folder to monitor and previewing virtual slides. The stitching module handles sequential stitching and image composition. The controller module manages the data exchange between the GUI and stitching modules and monitors file creation events in the specified folder. The major external libraries used in the system include OpenCV (4.5.1) [18] and Ceres Solver (version 2.0) [19]. Basic stitching processes such as feature point extraction and matching based on those features are implemented using OpenCV functionalities. However, several modifications were made to better fit the requirements of RT-4M. These include adding a translation-only constraint to affine transformation matrix computation, modifying the bundle adjustment process to incorporate distortion correction, and integrating with Ceres Solver for computational efficiency.

### 2.2. Details of Registration Stage

**Figure 2:**
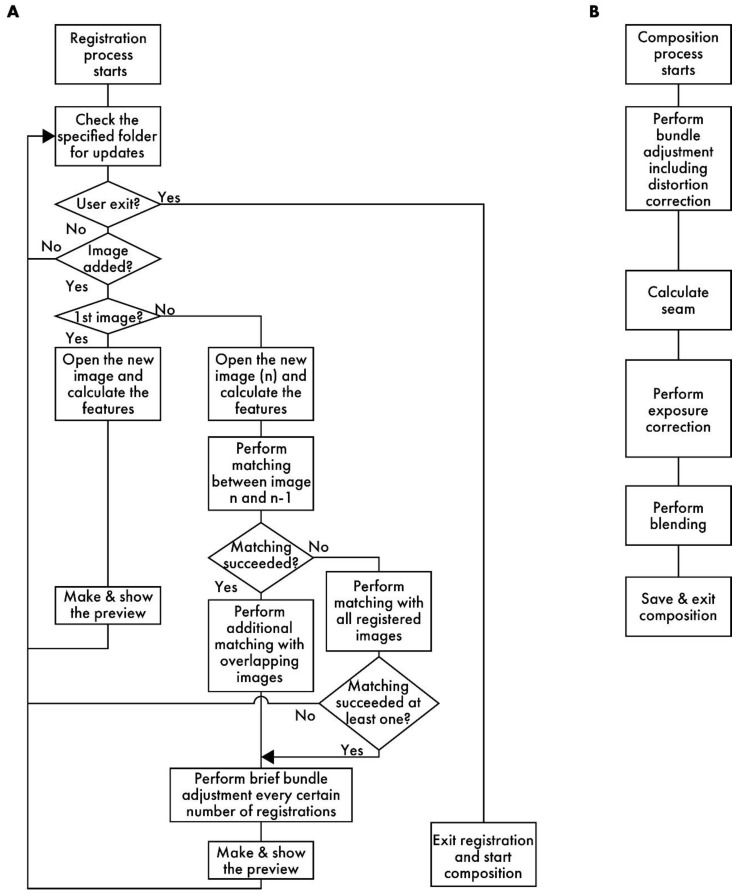
A flowchart of RT-4M. (**A**) Registration process: RT-4M monitors the specified folder, automatically loading the captured image files and performing stitching processes, such as feature point extraction, matching, and brief bundle adjustment, followed by displaying a preview. By repeating the imaging process, the user creates the virtual slide. (**B**) Composition process: This process includes image quality enhancement procedures, such as distortion correction, and saving the virtual slide.

Figure 2A presents a flowchart of the registration process. RT-4M monitors the folder specified by the user and detects the addition of image files to it. When a file is added, RT-4M reads it and extracts its features. RT-4M supports algorithms such as SIFT [20], AKAZE [21], ORB [22], BRISK [23], and KAZE [24] implemented in OpenCV for feature extraction. In this study, SIFT is used for virtual slide creation because preliminary experiments demonstrate that it exhibits sufficient speed and accuracy for hematoxylin and eosin (HE)- and fluorescent-stained samples. If the read image is the first one, it is displayed on the preview screen, and the monitoring of the folder continues. For the n-th image (*n* > 1), feature point matching is performed with the (*n* − 1)-th image. This yields an affine transformation matrix between the two images, which is used to project the n-th image onto the global coordinate system (i.e., coordinate system of the virtual slide being created). The positional relationships between the n-th image and the first (*n* − 2) images are also calculated. If matching between the *n*-th and (*n* − 1)-th images is unsuccessful, matching is re-attempted with all previous images. This allows virtual slide creation to continue from a new position as long as the *n*-th image overlaps with any previously captured image, even if the user captures an image far from the immediately preceding FOV. This is useful because, even though operating RT-4M fundamentally involves maintaining overlaps with the previous image while capturing each new image, this continuity cannot always be maintained when capturing a wide region. For example, if imaging ends at the edge of the sample and resumes from another uncaptured area, RT-4M attempts matching with all previous images to handle such cases, thereby improving the flexibility of virtual slide creation.

Based on the positional relationships obtained via matching, images overlapping with the n-th image are identified, and additional matching is performed with them. By filtering the matching target images based on positional relationships, the computation time is reduced. Subsequently, brief bundle adjustments are performed at predetermined intervals. Although bundle adjustment is a term usually used in 3D image reconstruction, we utilize it in the context of 2D stitching to denote an operation that reduces the reprojection errors of matched feature point pairs. RT-4M uses the Levenberg–Marquardt method [25] for bundle adjustment, which minimizes errors by iterating the following equation:

(1) $$x_{i+1}=x_{i}-{\left(J_{i}^{T}J_{i}+\lambda I\right)}^{-1}J_{i}^{T}\;r_{i}$$

Here, **x***_i_* denotes a vector comprising the parameters of the affine transformation matrix used for projecting each image onto global coordinates (*i* represents the iteration number). RT-4M is restricted to either translation (parameters: *a* and *b*), translation and rotation (parameters: *a*, *b*, and *θ*), or translation, rotation, and scaling (parameters: *a*, *b*, *θ*, and *s*). ***J****_i_* denotes the Jacobian matrix ($J_{i}^{T}$: the transpose of ***J****_i_*), **r***_i_* denotes the vector of the reprojection errors of the feature point pairs, *λ* denotes the damping scalar parameter adjusted at each iteration, and ***I*** denotes the identity matrix. Ceres Solver is used for implementation. During the registration process, a few iterations of Equation (1) are computed after a specified number of registrations to reduce errors. In this study, one iteration is computed after every ten registrations. By performing additional matching and brief bundle adjustments in this manner, RT-4M maintains real-time performance without hindering the microscope imaging operations of the user while simultaneously eliminating errors accumulated from sequential stitching. Finally, a preview image is generated and presented to the user. The registration process repeats these steps, synchronizing with the user’s imaging actions to execute stepwise stitching, thereby enabling real-time virtual slide creation.

### 2.3. Details of Composition Stage

Figure 2B depicts a flowchart of the composition process. First, bundle adjustment is executed. Unlike the brief bundle adjustment during the registration process, iterations are computed until the error converges. Moreover, by adding distortion parameters to the bundle adjustment parameters, a more precise correction is executed. Distortions caused by optical systems, such as microscope lenses, are known; on this basis, the true coordinates *x*, *y*, and *z* (scalars representing spatial positions) and the distorted coordinates *u* and *v* (scalars representing image coordinates) are approximated using the following equations [18,26]:

(2) $$x^{\prime }=x/z\;y^{\prime }=y/z\;r^{2}=x^{\prime 2}+y^{\prime 2}\;x^{\prime \prime }=x^{\prime }\frac{1+k_{1}r^{2}+k_{2}r^{4}+k_{3}r^{6}}{1+k_{4}r^{2}+k_{5}r^{4}+k_{6}r^{6}}+2p_{1}x^{\prime }y^{\prime }+p_{2}(r^{2}+2x^{\prime 2})\;y^{\prime \prime }=y^{\prime }\frac{1+k_{1}r^{2}+k_{2}r^{4}+k_{3}r^{6}}{1+k_{4}r^{2}+k_{5}r^{4}+k_{6}r^{6}}+p_{1}(r^{2}+2y^{\prime 2})+p_{2}x^{\prime }y\prime \;u=f_{x}x^{\prime \prime }+c_{x}\;v=f_{y}y^{\prime \prime }+c_{y}$$

Here, *k*_1_, *k*_2_, *k*_3_, *k*_4_, *p*_1_, and *p*_2_ denote the distortion coefficients; *f_x_* and *f_y_* denote the focal lengths; and *c_x_* and *c_y_* denote the principal point coordinates. These are all scalar entities. During registration, the parameter vector **x***_i_* in Equation (1) consists of only the parameters associated with the affine transformation matrix for each image. However, during composition, distortion parameters are added to estimate the distortion caused by the optical system. As the optical system of the microscope is usually stable and distortion parameters rarely vary between images, RT-4M sets common distortion parameters for all images. This prevents an increase in the number of parameters in the computationally intensive bundle adjustment process. RT-4M performs the inverse of the transformation given by the above equations to iterate Equation (1) using numerical calculations based on Newton’s method in the absence of an analytical solution for the inverse transformation. Subsequently, well-known stitching processes, such as seam calculation, exposure correction, and blending, are performed [6]. These are executed using OpenCV. For large virtual slides, these processes are executed by dividing them into appropriate sizes to mitigate memory constraints. During performance evaluation, exposure correction and blending processing are intentionally turned off to make the alignment errors more apparent.

### 2.4. Sample Preparation

All samples were stained using standard histological methods [27]. HE staining was performed by deparaffinizing and hydrating formalin-fixed paraffin-embedded (FFPE) sections, followed by staining with Mayer’s hematoxylin and 0.5% eosin Y ethanol (FUJIFILM Wako Pure Chemical Corp., Osaka, Japan). Fluorescence staining was performed by deparaffinizing and hydrating FFPE sections, followed by staining with Alexa 488 phalloidin (1:200 dilution) and Hoechst (1:500 dilution) (Thermo Fisher Scientific Inc., Waltham, MA, USA). Human liver FFPE sections were purchased from Origene Technologies, Inc. (Rockville, MD, USA). FFPE sections of various mouse tissues were purchased from Genostaff Co., Ltd. (Tokyo, Japan). Human pancreatic cancer FFPE sections were obtained from the Tsukuba Human Tissue Biobank Center (Tsukuba, Ibaraki, Japan).

### 2.5. Imaging

The stained samples were imaged using an inverted bright-field and fluorescence microscopy system (microscope: Eclipse Ts2R; camera: DS-Ri2; image acquisition software: NIS-Elements (5.10), all purchased from Nikon Corp. (Tokyo, Japan)) or an upright bright-field microscopy system (microscope: BX51WI (Evident Corp., Tokyo, Japan); camera: EOS Kiss X7 (Canon Inc., Tokyo, Japan); image acquisition software: EOS Utility (Canon Inc.)) (Figure 1B,C, Table 1). RT-4M was installed on the camera control PC running the image acquisition software. For control experiments, imaging was also performed using automated microscopy (BZ-9000, KEYENCE Corp., Osaka, Japan) and a slide scanner (NanoZoomer S360, Hamamatsu Photonics K.K., Shizuoka, Japan).

### 2.6. Performance Evaluation

To evaluate registration performance, the user’s microscope imaging operation time was disregarded, and sequential stitching was performed using pre-captured images using the imaging equipment mentioned in Section 2.5. Additionally, for comparison with existing software, virtual slides were created using the ImageJ (1.54p) stitching plugin [11] and MicroMos (3.0.2) [10]. The ImageJ stitching plugin was used in both the sequential and unknown modes. The sequential mode stitches images by assuming an overlap between adjacent images in the list. The unknown mode stitches images without assuming any positional relationship; instead, it explores potential overlaps between all image pairs. A PC running Windows 10, with an Intel Xeon E-2186G 3.80GHz processor and a 32 GB RAM, was used for performance evaluation. The performance evaluation results were statistically tested using the Wilcoxon signed-rank test. The *p*-values of multiple comparisons were corrected using the Benjamini–Hochberg method.

## 3. Results

### 3.1. Creation of Virtual Slides of Various Samples

**Figure 3:**
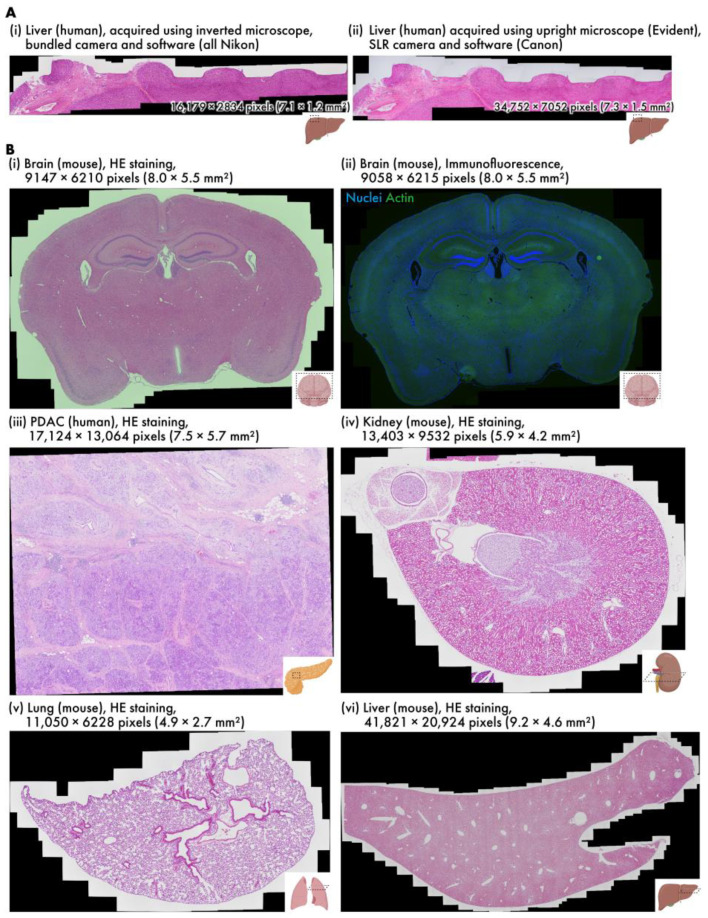
Various virtual slides created using RT-4M. (**A**) HE-stained human liver samples captured using different devices (h-liver-HE, h-liver-HE-canon). (**B**) Virtual slides of different tissues: (**i**) HE-stained mouse brain (m-brain-HE), (**ii**) double fluorescent-stained mouse brain (m-brain-IF), (**iii**) HE-stained human PDAC (h-pdac-mod-HE), (**iv**) HE-stained mouse kidney (m-kidney-HE), (**v**) HE-stained mouse lung (m-lung-HE), (**vi**) HE-stained mouse liver (m-liver-HE). Dataset names are provided within parentheses.

Virtual slides of various samples are created using RT-4M (Table 1, Video S1). The wait time is observed to be insignificant in all cases; thus, the user’s microscope imaging operation is not hindered. Additionally, as imaging is performed simultaneously with the constant confirmation of the preview screen, no unintentional losses are observed in the virtual slides, and no reimaging is required. Figure 3A depicts virtual slides of HE-stained human liver sections created using different equipment. The sample depicted in Figure 3A(i) was captured using an inverted bright-field and fluorescence microscopy system composed entirely of genuine Nikon parts, and that in Figure 3A(ii) was captured with an upright bright-field microscopy system equipped with an Evident microscope and a Canon SLR camera. RT-4M is operated without any issues, and equivalent virtual slides are generated for both microscopy systems.

Figure 3B depicts virtual slides created using an inverted bright-field and fluorescence microscopy system. Figure 3B(i) depicts an HE-stained mouse brain section (dataset: m-brain-HE), and Figure 3B(ii) depicts a fluorescent-stained mouse brain section (dataset: m-brain-IF). Figure 3B(ii) depicts a two-color image, where each color is captured by switching the wavelength filter of the microscope. The exposure time for bright-field imaging is 0.5 ms, whereas that for fluorescence imaging is 400 ms when averaged over four iterations, requiring approximately 1.6 s per color. RT-4M is observed to create virtual slides in these cases without any issues. This indicates that RT-4M can handle not only bright-field imaging, such as HE staining, but also fluorescent imaging, which requires filter switching and long exposure times. Figure 3B(iii) presents an HE-stained image of human pancreatic ductal adenocarcinoma (PDAC) (dataset: h-pdac-mod-HE). Although the sample contains regions with densely packed cells and those primarily composed of glandular structures with sparse tissue, RT-4M successfully creates virtual slides of the samples notwithstanding their varying characteristics. Figure 3B(iv,v) depict HE-stained images of a mouse kidney and lung, respectively (datasets: m-kidney-HE, m-lung-HE). These tissues are sparser than those presented in (i)–(iii), but RT-4M performs stitching accurately even in these cases. In addition, Dataset (v) does not maintain continuity, i.e., during the creation of the virtual slide, several images are captured at positions without overlaps with previous images. RT-4M performs matching with all previously captured images when no overlap is detected, allowing it to handle such cases. Figure 3B(vi) illustrates an HE-stained image of a mouse liver (dataset: m-liver-HE). The liver exhibits extremely homogeneous tissue, and feature extraction and matching are prone to failure under such conditions. Moreover, the virtual slide is constructed based on the largest number of images among the datasets in this study, with a total of 908 images and a final pixel count of 41,821 × 20,924 pixels, without imaging continuity. Despite these severe conditions, RT-4M is observed to yield an accurate virtual slide.

### 3.2. Comparison with Automated Microscopy and Slide Scanner

**Figure 4:**
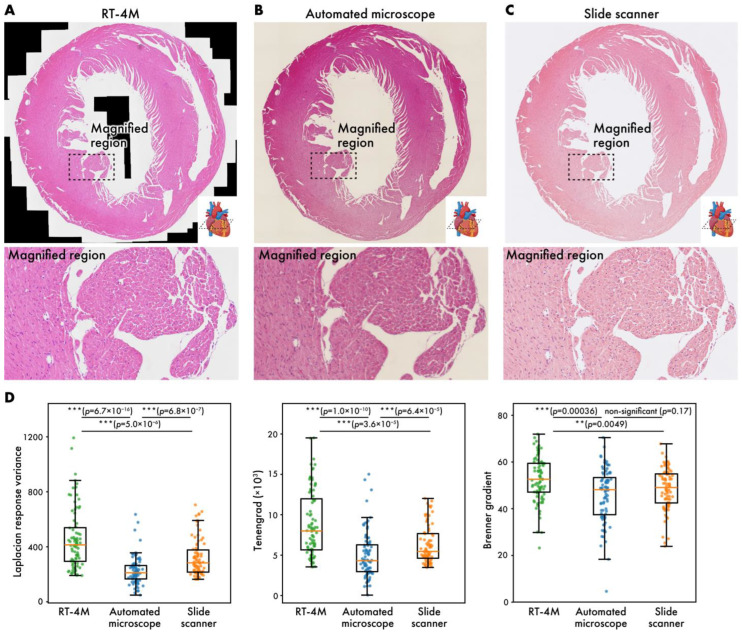
Virtual slides of HE-stained mouse heart (m-heart-HE) created using different systems. (**A**) RT-4M, (**B**) automated microscope, (**C**) slide scanner. (**D**) Comparison of virtual slide sharpness across imaging methods. Sharpness was evaluated by randomly sampling 85 small regions on each virtual slide and calculating Laplacian response variance, Tenengrad, and Brenner gradient. Statistical analysis was performed using Wilcoxon rank-sum test. *p*-values of multiple comparisons were corrected using Benjamini–Hochberg method.

The current gold standard for creating virtual slides involves automated microscopy or the use of slide scanners. In this subsection, virtual slides of HE-stained mouse heart sections (dataset: m-heart-HE) created using RT-4M are compared with those created using the aforementioned systems (Figure 4). Despite color differences arising from camera configurations, the virtual slides created using RT-4M do not exhibit any obvious deformation or defects compared to the other two virtual slides. Enlargements of the images also do not reveal any deformations or defects, indicating that RT-4M creates virtual slides with similar accuracy compared to automated microscopes and slide scanners. Although this does not affect observation, owing to the stitching mechanism based on image overlap, large cavities such as ventricles may appear as blanks (indicated using black regions) in RT-4M-created virtual slides. We compared the image quality produced by each imaging method by randomly sampling 85 small regions on the virtual slides and computing three well-established sharpness metrics [28,29], Laplacian response variance, Tenengrad, and Brenner gradient, for evaluation (Figure 4D). For these calculations, the RT-4M and slide scanner virtual slides were down-sampled to match the lowest-resolution instrument, the automated microscope. Across all three metrics, the virtual slides acquired with RT-4M were equal to or better than those obtained with the automated microscope or slide scanner. Because RT-4M virtual slides were captured using a bio-research-grade microscope without any quality-degrading processing, and focus was manually adjusted by visual inspection during acquisition, RT-4M likely achieved sharpness equal or superior to those of the other imaging methods.

### 3.3. Performance Evaluation of Additional Matching During Registration

**Figure 5:**
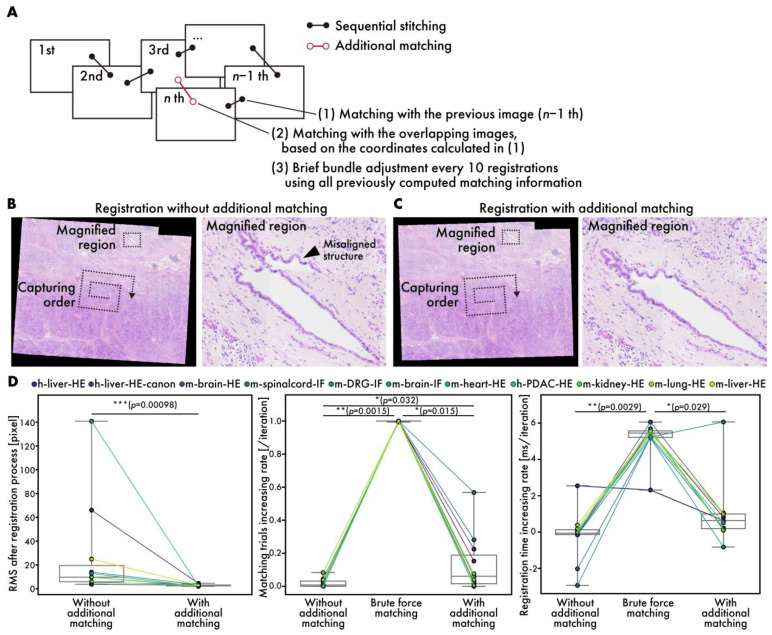
Performance evaluation of additional matching. (**A**) Schematic diagram showing additional matching process. (**B**,**C**) HE-stained human PDAC virtual slides (h-pdac-HE) (**B**) without and (**C**) with additional matching. Arrowheads indicate significantly misaligned structures. (**D**) Quantitative performance evaluation: comparison of RMS values at end of registration process (**left**), comparison of rates of increase in matching trials (**middle**), and comparison of rates of increase in registration time (**right**). Plot colors represent different datasets, with points corresponding to same dataset connected by lines. Statistical analysis is performed using Wilcoxon signed-rank test. *p*-values of multiple comparisons are corrected using Benjamini–Hochberg method.

Generally, sequential stitching can lead to cumulative errors as the number of images is increased [14]. RT-4M addresses this issue by performing additional matching, including brief bundle adjustments, to reduce cumulative error (Figure 5A). Figure 5B,C depict virtual slides during registration with and without additional matching. Owing to the capture of data (h-mod-pdac-HE) in a clockwise spiral pattern starting from the center while maintaining continuity, cumulative errors are prone to occurring towards the periphery. The enlargement of the peripheral areas reveals significant misalignment in the absence of additional matching (Figure 5A). In contrast, with additional matching, no significant misalignment is observed. The root mean square (RMS) errors at the end of the registration process with and without additional matching are 27 and 2.9 pixels, respectively, indicating that additional matching reduces the error significantly (Figure 5D). Particularly for datasets with large initial errors, e.g., h-pdac-mod-HE and h-liver-HE, the RMS values decrease from 140 to 2.5 and 66 to 4.5 pixels, respectively, demonstrating the effectiveness of additional matching. The RMS is defined as the root mean square of the reprojection errors computed over all matched point pairs as follows:

$$\mathrm{RMS}=\sqrt{\frac{1}{N}\sum_{i=1}^{N}{\left\|p_{i}^{\left(1\right)}\left.-p_{i}^{\left(2\right)}\right\|\right.}^{2}}$$

Here, $p_{i}^{\left(1\right)}$ and $p_{i}^{\left(2\right)}$ denote the reprojected global coordinates of the i-th matched point pair; N is the total number of matched point pairs; and $\left\|\;\right.\left.\cdot \;\right\|$ denotes the Euclidean norm. To evaluate the effects of additional matching on the user’s wait time, the rates of increase in the number of matching trials per image registration and the time required to register one image are measured (Figure 5D, Figures S1 and S2). The brute force matching method is considered as the worst case, in addition to the conditions with and without additional matching. The matching trials and registration times are fitted linearly with respect to the number of registration iterations, and the slopes are considered to represent the rates of increase. The number of matching trials does not increase without additional matching, while it does by 1 per iteration when brute force matching is used, as expected. In contrast, with additional matching, the rate of increase is observed to be 0.13 per iteration on average. The rate of increase is higher on datasets with fewer images and lower than 0.1 per iteration on datasets with over 80 images, where the number of matching trials remains almost constant (Figure S1). This is expected given the mechanism of filtering in matching targets based on positional relationships. The rate of increase in registration time per iteration is observed to be 0.19 ms/iteration without additional matching, 5 ms/iteration with brute force matching, and 0.97 ms/iteration with additional matching on average over all datasets. The final registration time on the largest dataset, m-liver-HE, is 0.55 s without additional matching, 5.17 s with brute force matching, and 0.81 sec with additional matching. Users typically spend 3–4 s adjusting the FOV and focus before capturing an image; the brute force registration time exceeds this timeframe and could affect the user’s imaging operation. On the other hand, the rate of increase with additional matching is sufficiently small, fitting well within this timeframe, even during the final registration on the large dataset consisting of more than 900 images. Although brief bundle adjustment is executed once every ten iterations and requires more time than normal registration, the registration time, including brief bundle adjustment, is approximately 4 s, even for the 900-th image, which is considered acceptable to users.

### 3.4. Performance Evaluation of Distortion Correction in Composition Stage

**Figure 6:**
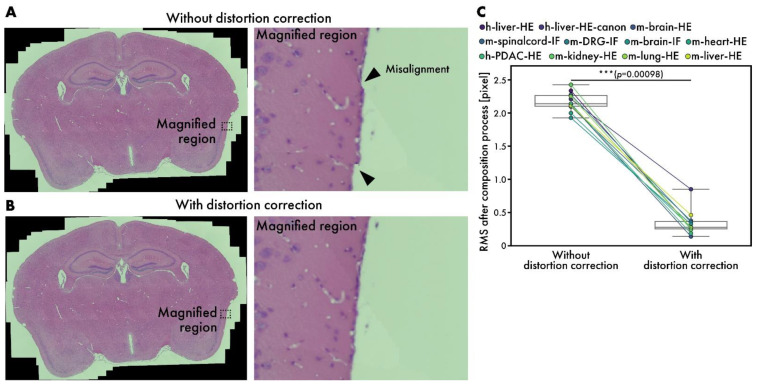
Performance evaluation of distortion correction. (**A**,**B**) HE-stained mouse brain virtual slides (m-brain-HE) (**A**) without and (**B**) with distortion correction. Arrowheads indicate misalignments. (**C**) Comparison of RMS values at end of composition process with and without distortion correction. Plot colors represent different datasets, with points corresponding to same dataset connected by lines. Statistical analysis is performed using Wilcoxon signed-rank test. *p*-values of multiple comparisons are corrected using Benjamini–Hochberg method.

Although microscopes are well-aligned optical instruments, slight distortions may occur sometimes. While stitching many images, the errors caused by these distortions accumulate, leading to visible misalignments, as depicted in Figure 6A. By introducing distortion correction, RT-4M corrects misalignments to an imperceptible level (Figure 6B); Figure 6B zooms in on the areas corresponding to misalignment in Figure 6A. Considering the possibility of misalignment in other parts owing to distortion correction, the entire peripheral area is also inspected, and no misalignment is observed. After the composition process, the average RMS values are 2.17 pixels without distortion correction and 0.34 pixels with it, indicating the effectiveness of the subpixel-level correction of misalignment (Figure 6C).

### 3.5. Comparison with Other Software

To evaluate the quality of the created virtual slides, comparisons are performed with existing software, ImageJ stitching plugin and MicroMos. The ImageJ stitching plugin is used in both the sequential and unknown modes. Although other stitching software is available, most assume a grid format for image acquisition or employ methods that make no assumptions about image positional relationships, rendering them unsuitable for continuous images with arbitrary overlap; therefore, we opted to compare these two readily accessible software packages. The ImageJ unknown mode is a technique that makes no assumptions about the positional relationships between images, in contrast to the ImageJ sequential mode, MicroMos, and RT-4M. However, it was included in our comparative evaluation for reference. Of note, because MicroMos encounters errors while processing full-size images, virtual slides are created from half-sized images. Visual evaluation is performed to assess quality as programmatic evaluation is difficult. First, entire virtual slides are checked for noticeable stitching failures. The stitching result is considered “success” if no visibly missing tiles or obviously misaligned tiles stitched into incorrect positions are observed. If any tile is clearly missing or stitched into a visibly incorrect location, the result is marked as “fail”. In the sequential mode, ImageJ fails to stitch m-lung-HE and m-liver-HE, whereas in the unknown mode, it fails to stitch h-liver-HE, h-liver-HE-canon, m-brain-HE, m-brain-IF, m-heart-HE, and m-kidney-HE. The m-liver-HE data from ImageJ in unknown mode are missing due to the non-completion of the program. MicroMos fails to stitch m-kidney-HE, m-lung-HE, and m-liver-HE. The failures of ImageJ in sequential mode and MicroMos in stitching m-lung-HE and m-liver-HE are expected because these datasets do not maintain continuity. In contrast, no failure is observed on any of the datasets when RT-4M is used. This is because RT-4M recovers from registration failures by performing exhaustive matching with all previously registered images when matching with the immediately preceding image fails. As such, it is more robust than other methods that rely solely on simple sequential registration. Furthermore, we compared the total processing time to evaluate the speed performance of the software (Table 2). RT-4M demonstrated performance on par with the sequential mode of the ImageJ stitching plugin, which is highly popular in bioscience. In addition, it was faster than the unknown mode of the ImageJ Stitching plugin and MicroMos. The performance of the unknown mode was as expected, since this mode does not assume overlap between consecutive images. Notably, for the largest dataset (m-liver-HE), which contains 908 images, the ImageJ sequential mode required 13,338.7 s and ultimately failed to produce a valid stitching result, whereas RT-4M successfully completed stitching in 2183.4 s.

**Figure 7:**
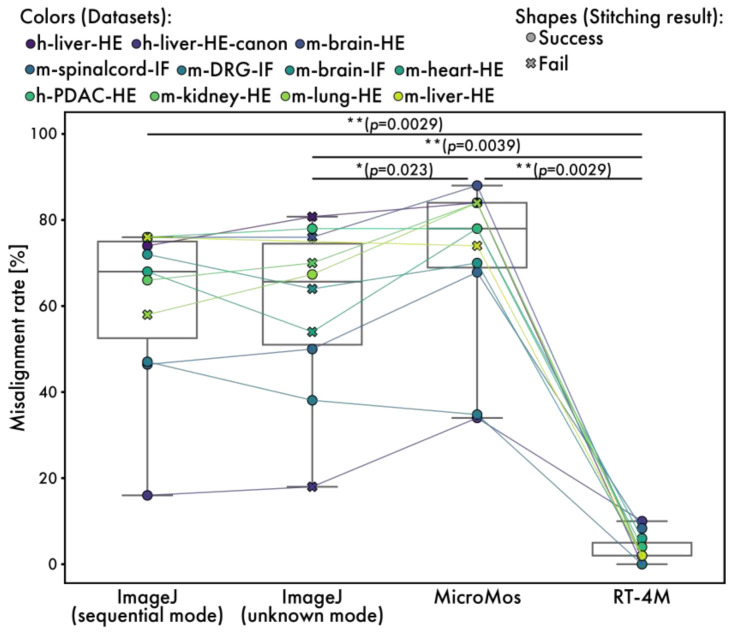
Measured misalignment rates for RT-4M and existing software. Plot colors represent different datasets, with points of the same dataset connected by lines. The misalignment rate indicates the proportion of randomly selected local regions in the virtual slide where a visually detectable misalignment of fine structures was observed (Figure 6A). The plot shape (circle and cross) indicates the stitching result, success and fail, respectively. The stitching result is defined based on a visual inspection of the entire stitched image. A result is considered “success” if no visibly missing tiles or obviously misaligned tiles stitched into incorrect positions are observed. If any tile is clearly missing or stitched into a visibly incorrect location, the result is marked as “fail”. Statistical analysis is performed using the Wilcoxon signed-rank test. The *p*-values of multiple comparisons are corrected using the Benjamini–Hochberg method.

Next, a detailed evaluation of minor misalignments is performed by extracting 50 small regions randomly from each virtual slide and visually checking for noticeable stitching misalignments (Figure 7). The size of each small region is 512 × 512 pixels for ImageJ and RT-4M and 256 × 256 pixels for MicroMos, because the virtual slides created by MicroMos are downsized. If a virtual slide is too small to extract 50 regions, randomly extracted non-overlapping regions are used. The percentage of regions with visible misalignments is 61.4% (60.2%) for ImageJ in the sequential mode, 59.6% (58.4%) for ImageJ in the unknown mode, and 70.6% (66.8%) for MicroMos (values in parentheses exclude datasets with stitching failure) compared to 3.5% for RT-4M.

## 4. Discussion

This study proposes and evaluates RT-4M, a software for real-time virtual slide creation. By installing RT-4M on any manual microscopy system, users can create virtual slides in real time while capturing images simultaneously. The key design features that enable this functionality are a loosely coupled configuration with a camera and image acquisition software. This allows for compatibility with various existing microscopy systems. RT-4M is observed to create equivalent virtual slides using a relatively inexpensive configuration with an SLR camera attached to the microscope or a more expensive configuration with a dedicated microscope camera supplied by the manufacturer. This indicates that users can continue using their existing microscopy systems, enabling inexpensive virtual slide creation and high usability by seamlessly integrating them into familiar workflows. Another critical design feature of RT-4M is its two-stage workflow, consisting of a registration process that performs real-time sequential stitching and preview generation and a composition process that performs distortion correction to improve output quality. Registration maintains real-time performance while reducing significant misalignments that lead to missed captures by performing additional matching and brief bundle adjustments. This allows the entire tissue area to be captured without omissions, even when creating virtual slides consisting of more than 900 images within a reasonable wait time. Capturing 900 images is considered to be the upper limit in terms of workload while creating virtual slides using a manual microscope. Assuming that capturing a single image takes approximately 4 s, including adjustments for FOV and focus, capturing 900 images would require continuous operation for one hour. Therefore, RT-4M is considered to exhibit satisfactory performance for practical applications. During the composition process, distortion correction is introduced into the bundle adjustment to correct optical system distortions, achieving subpixel-level RMS values. RT-4M demonstrated a speed performance comparable to the ImageJ stitching plugin widely used in bioscience and outperformed it on large datasets. In addition, RT-4M achieved a nearly 20-fold reduction in the occurrence rate of visually detectable misalignments compared with existing software, a benefit presumed to arise from its distortion correction feature.

Although RT-4M allows users to create virtual slides cheaply, easily, and accurately using existing microscopy systems, it exhibits certain limitations. First, the loosely coupled architecture involving the camera and image acquisition software via storage is slower than on-memory real-time methods. In virtual slide creation with RT-4M, the process comprises primarily field-of-view movement and image capture, including file saving time. Empirically, image capture including file saving accounts for approximately 50% of the total acquisition time. Because on-memory real-time methods eliminate the need for explicit user-driven capture operations, they are expected to be more than twice as fast. This is a trade-off with versatility and compatibility with fluorescent samples. However, despite being slower, RT-4M exhibits sufficient real-time performance to follow imaging operations using manual microscopes, making it suitable for creating small-to-medium-sized virtual slides. Second, additional matching during the registration process results in slightly longer wait times every ten iterations. Although this is within the usual imaging operation timeframe, skilled operators may find it stressful. The frequency of additional matching was not optimized in this study and is empirically set to once every ten iterations. Ideally, this frequency should be determined adaptively based on the imaging equipment and sample type used during imaging. Finally, during the composition process, RT-4M assumes the principal point of the distortion parameters to be at the center of the image. Although this assumption is typically valid for microscopy, it may fail in some cases.

Existing methods for creating virtual slides can be broadly categorized into three types: (i) using automated microscopes or slide scanners, (ii) capturing images with a manual microscope and stitching them using software (offline method), and (iii) dynamically reading images from the camera and stitching them while moving the microscope manually (on-memory real-time method). Methods of type (i) can create large quantities of virtual slides quickly but represent an expensive solution. RT-4M is not intended to replace such systems; it is designed for scenarios where routine observation operations with a manual microscope lead to the creation of partial virtual slides or for small-to-medium-sized laboratories or hospitals that cannot afford such equipment. Therefore, comparisons with methods of types (ii) and (iii) are more relevant for potential RT-4M users. Methods of type (ii), which are offline methods, allow for the use of existing microscopy systems and are easily adaptable to fluorescent samples. However, they lack real-time preview capabilities during imaging and risk the need for reimaging owing to missed captures or stitching errors. Methods of type (iii), i.e., on-memory real-time methods, provide real-time preview capabilities during imaging, reducing reimaging costs; however, they are challenging to adapt for fluorescent samples and require dedicated cameras, hindering their implementation using existing cameras and image acquisition software. RT-4M satisfies all of the aforementioned criteria—using existing microscopy systems, providing real-time preview capabilities, and supporting fluorescent samples.

In conclusion, we believe that RT-4M is an important software for creating virtual slides using manual microscopes. RT-4M has been released as free software in the repository and is expected to be used in small- and medium-sized facilities with limited budgets. Future developments should focus on enhancing the interface and addressing the limitations of this study.

## Figures and Tables

**Table 1:** Description of datasets.

Dataset Name	Species	Tissue	Imaging Method	Microscope	Camera	Exposure (ms)	Averaging	Number of Images	Dimension (Pixels)	Continuity
h-liver-HE	Human	Liver	Bright field	Nikon Eclipse Ts2R	Nikon DS-Ri2	7	1	59	1636 × 1088	Yes
h-liver-HE-canon	Human	Liver	Bright field	Evident BX51WI	Canon EOS Kiss X7	1	1	37	5184 × 3456	Yes
m-brain-HE	Mouse	Brain	Bright field	Nikon Eclipse Ts2R	Nikon DS-Ri2	0.5	1	154	1636 × 1088	Yes
m-spinalcord-IF	Mouse	Spinal cord	Fluorescence	Nikon Eclipse Ts2R	Nikon DS-Ri2	400	1	25	1636 × 1088	Yes
m-DRG-IF	Mouse	DRG	Fluorescence	Nikon Eclipse Ts2R	Nikon DS-Ri2	400	4	15	1636 × 1088	Yes
m-brain-IF	Mouse	Brain	Fluorescence	Nikon Eclipse Ts2R	Nikon DS-Ri2	400	4	90	1636 × 1088	Yes
m-heart-HE	Mouse	Heart	Bright field	Nikon Eclipse Ts2R	Nikon DS-Ri2	1	1	164	1636 × 1088	Yes
h-pdac-mod-HE	Human	PDAC	Bright field	Nikon Eclipse Ts2R	Nikon DS-Ri2	1	1	320	1636 × 1088	Yes
m-kidney-HE	Mouse	Kidney	Bright field	Nikon Eclipse Ts2R	Nikon DS-Ri2	0.4	1	164	1636 × 1088	Yes
m-lung-HE	Mouse	Lung	Bright field	Nikon Eclipse Ts2R	Nikon DS-Ri2	0.4	1	80	1636 × 1088	No
m-liver-HE	Mouse	Liver	Bright field	Nikon Eclipse Ts2R	Nikon DS-Ri2	1	1	908	1636 × 1088	No

**Table 2:** A comparison of the total processing time between the existing software and RT-4M. The total processing time indicates the time from image registration to file output without user operation. Instances where the stitching result was classified as “fail” are indicated in parentheses. The stitching result is defined based on a visual inspection of the entire stitched image. A result is considered “success” if no visibly missing tiles or obviously misaligned tiles stitched into incorrect positions are observed. If any tile is clearly missing or stitched into a visibly incorrect location, the result is marked as “fail”.

Dataset	Total Processing Time of Each Software (s)			
	ImageJ Sequential Mode	ImageJ Unknown Mode	MicroMos	RT-4M
h-liver-HE	46.2	957.4 (Fail)	426.0	51.3
h-liver-HE-canon	226.9	2946.1 (Fail)	1170.0	331.8
m-brain-HE	177.4	6750.7 (Fail)	1260.0	161.0
m-spinalcord-IF	19.9	152.5	110.0	38.9
m-DRG-IF	12.4	50.2	57.0	30.0
m-brain-IF	66.5	1886.7 (Fail)	713.0	111.0
m-heart-HE	119.5	6932.0 (Fail)	2312.0	177.3
h-pdac-mod-HE	236.1	22,806.3	6671.0	561.1
m-kidney-HE	137.8	6312.6 (Fail)	2225.0 (Fail)	209.3
m-lung-HE	60.4 (Fail)	1461.9	618.0 (Fail)	117.7
m-liver-HE	13,338.7 (Fail)	(non-completion)	21,414.0 (Fail)	2183.4

## Data Availability

The software RT-4M is available at https://github.com/mori-nobuhito/rt4m-pub/ (accessed on 1 April 2025). The image dataset is available from the corresponding author upon reasonable request. Further inquiries can be directed to the corresponding authors.

## Supplementary Materials

The following supporting information can be downloaded at https://www.mdpi.com/article/10.3390/s25102968/s1, Figure S1: Measured trend in registration iteration number with respect to number of matching trials on each dataset; Figure S2: Measured trends in registration time with respect to number of matching trials on each dataset; Video S1: A movie demonstrating the operation of RT-4M.

## Abbreviations

The following abbreviations are used in this manuscript:

FFPE	Formalin-fixed paraffin-embedded
FOV	Field-of-view
HE	Hematoxylin and eosin
PDAC	Pancreatic ductal adenocarcinoma
RMS	Root mean square
RT-4M	Real-time mosaicing manager for manual microscopy system
