# Supplementary material for: RT-4M: Real-Time Mosaicing Manager for Manual Microscopy System
Source: Sensors (Basel). 2025 May 8;25(10):2968. doi: 10.3390/s25102968 (PMC12114642; doi:10.3390/s25102968)
Supplement: Supplementary file 1 [file sensors-25-02968-s001.zip › Supplementary_file.pdf]

sensors

MDPI

Supplementary Material

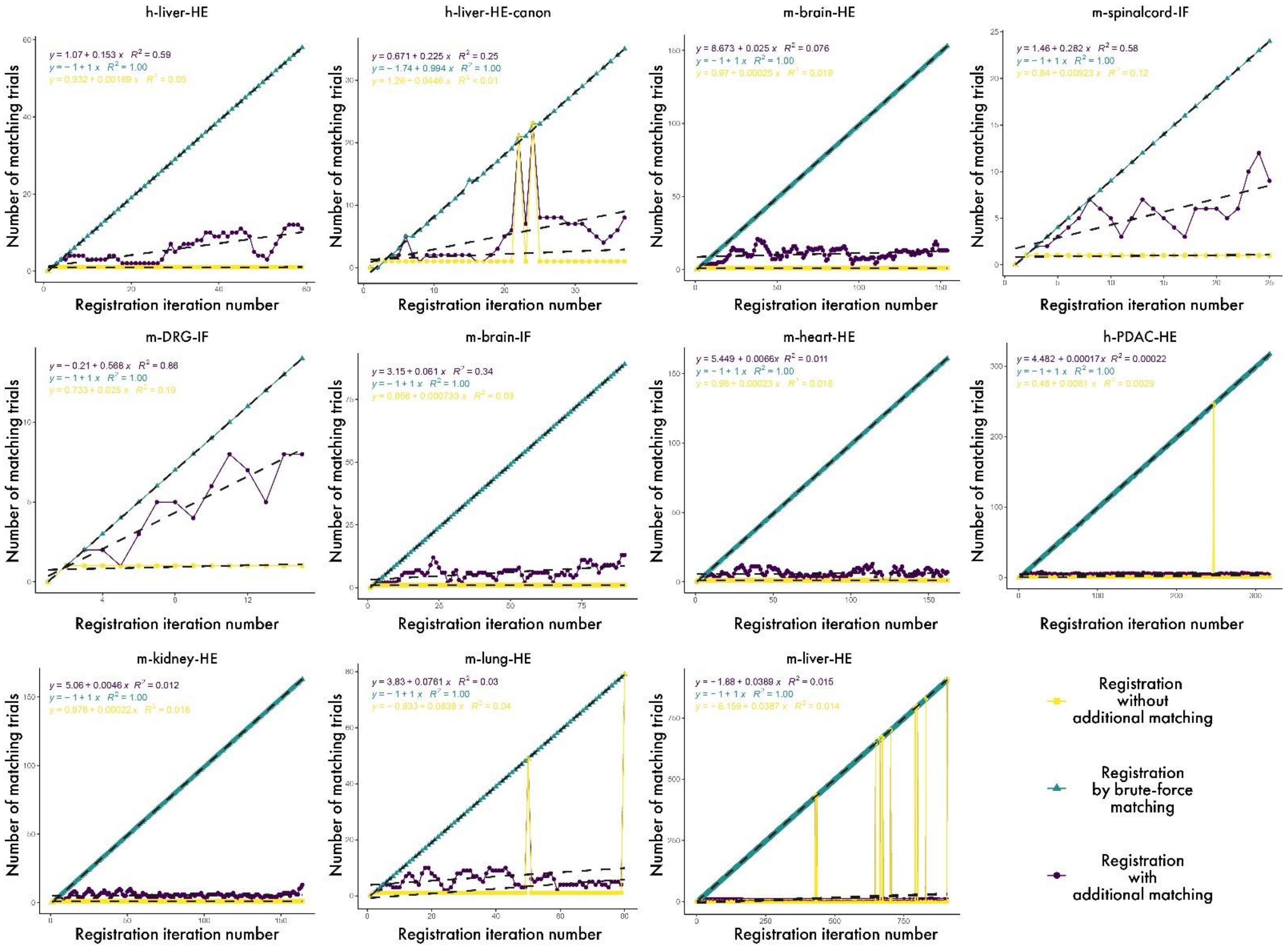

Figure S1. Measured trend of registration iteration number with respect to number of matching trials on each dataset.

Yellow square plots indicate registration without additional matching, green triangle plots indicate registration by brute force matching, and purple circle plots indicate registration with additional matching. The black dashed line represents the fitted line.

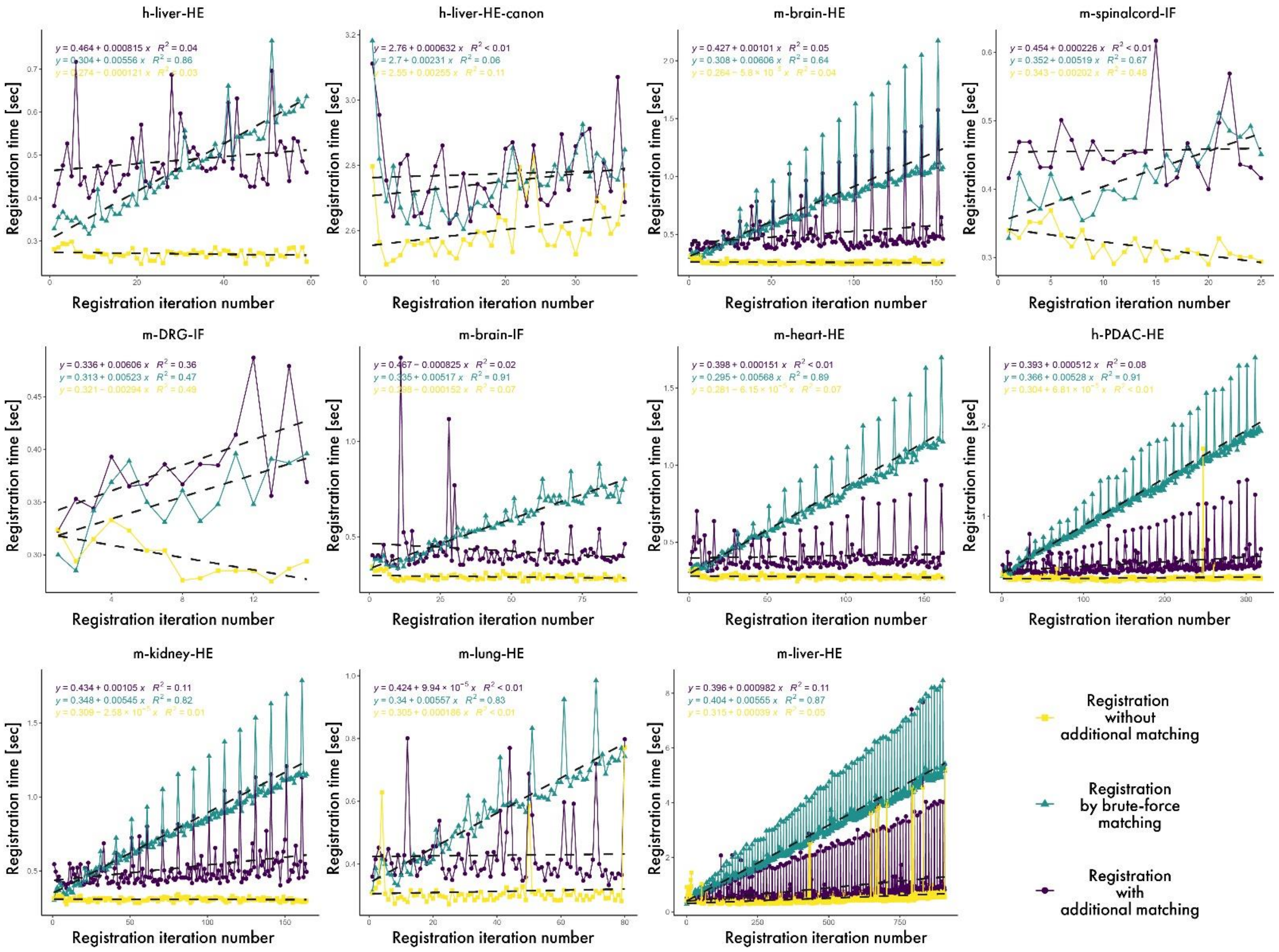

Figure S2. Measured trends of registration time with respect to number of matching trials on each dataset.

Yellow square plots indicate registration without additional matching, green triangle plots indicate registration by brute force matching, and purple circle plots indicate registration with additional matching. The black dashed line represents the fitted line.

Video S1. A movie demonstrating the operation of RT-4M.

The user captures images using the image acquisition software (left). RT-4M (right) detects the saved images, automatically loads them, and performs real-time stitching. The user can preview this process and create a virtual slide by gradually changing the field of view and repeatedly capturing images. The video speed is adjusted for demonstration.
